# Analysis of Factors Lowering Sensitivity of Interferon-γ Release Assay for Tuberculosis

**DOI:** 10.1371/journal.pone.0023806

**Published:** 2011-08-19

**Authors:** Nguyen Thi Le Hang, Luu Thi Lien, Nobuyuki Kobayashi, Takuro Shimbo, Shinsaku Sakurada, Pham Huu Thuong, Le Thi Hong, Do Bang Tam, Minako Hijikata, Ikumi Matsushita, Nguyen Van Hung, Kazue Higuchi, Nobuyuki Harada, Naoto Keicho

**Affiliations:** 1 NCGM-BMH Medical Collaboration Center, Hanoi, Viet Nam; 2 Hanoi Lung Hospital, Hanoi, Viet Nam; 3 Department of Respiratory Medicine, National Center for Global Health and Medicine, Tokyo, Japan; 4 Department of Clinical Research and Informatics, International Clinical Research Center, National Center for Global Health and Medicine, Tokyo, Japan; 5 Department of Respiratory Diseases, Research Institute, National Center for Global Health and Medicine, Tokyo, Japan; 6 Department of Biochemistry, Hematology and Blood Transfusion, Hanoi Lung Hospital, Hanoi, Viet Nam; 7 Department of Bacteriology, National Lung Hospital, Hanoi, Viet Nam; 8 Department of Mycobacterium Reference and Research, Research Institute of Tuberculosis, Tokyo, Japan; Fundació Institut Germans Trias i Pujol; Universitat Autònoma de Barcelona CibeRES, Spain

## Abstract

**Background:**

Imperfect sensitivity of interferon-**γ** release assay (IGRA) is a potential problem to detect tuberculosis. We made a thorough investigation of the factors that can lead to false negativity of IGRA.

**Methods:**

We recruited 543 patients with new smear-positive pulmonary tuberculosis in Hanoi, Viet Nam. At diagnosis, peripheral blood was collected and IGRA (QuantiFERON-TB Gold In-Tube) was performed. Clinical and epidemiological information of the host and pathogen was collected. The test sensitivity was calculated and factors negatively influencing IGRA results were evaluated using a logistic regression model in 504 patients with culture-confirmed pulmonary tuberculosis.

**Results:**

The overall sensitivity of IGRA was 92.3% (95% CI, 89.6%–94.4%). The proportions of IGRA-negative and -indeterminate results were 4.8% (95% CI, 3.1%–7.0%) and 3.0% (95% CI, 1.7%–4.9%). Age increased by year, body mass index <16.0, HIV co-infection and the increased number of HLA-DRB1*0701 allele that patients bear showed significant associations with IGRA negativity (OR = 1.04 [95% CI, 1.01–1.07], 5.42 [1.48–19.79], 6.38 [1.78–22.92] and 5.09 [2.31–11.22], respectively). HIV co-infection and the same HLA allele were also associated with indeterminate results (OR = 99.59 [95% CI, 15.58–625.61] and 4.25 [1.27–14.16]).

**Conclusions:**

Aging, emaciation, HIV co-infection and HLA genotype affected IGRA results. Assessment of these factors might contribute to a better understanding of the assay.

## Introduction

Tuberculosis (TB) remains a disease of serious concern; one third of the global population is infected with *Mycobacterium tuberculosis* (MTB) and eight to ten million people develop the disease every year [1]. The primary step to control TB is detecting infection by a sensitive test.

Recently, an immunoassay that measures interferon (IFN)-γ response to MTB-specific antigens (interferon-γ release assay; IGRA) has been developed. Studies on the use of IGRA in patients with active TB have had two purposes: (1) to evaluate performance of IGRA in latent TB infection (LTBI) using active TB as a surrogate, and (2) to determine whether IGRA plays a supplementary role in the exclusion of active TB disease in optimal setting [2]–[4].

IGRA use in diagnosis of LTBI has been established and supported by European and American guidelines [5], [6], whereas its use has not been recommended to rule out active disease particularly in high-burden countries, because of low sensitivity and low negative predictive values [7], [8]. Consequently, so far the sensitivity of IGRA varies from 64% to 92% [3], but the number of reports from high-burden countries is limited.

Imperfect sensitivity is a potential problem when using this assay to exclude LTBI as well as active TB. Due to the lack of a gold standard for LTBI identification, mechanisms by which IGRA gives false-negative results in LTBI are largely unknown [2], [3]. Identification and characterization of factors that lower the test sensitivity, by using active TB patients as a surrogate for LTBI suspects, would delineate active TB-disease specific and non-specific mechanisms that underlie false negative results of IGRA.

At present, however, there is no comprehensive report on relevant factors including extent of TB lesions, malnutrition, aging, HIV co-infection, and MTB strains. Inherent genetic variations are also candidate factors affecting IGRA results. Among these, polymorphism of human leukocyte antigen (HLA) is classically known to influence T-cell immune response and determines IFN-γ concentrations after stimulation with MTB antigens [9]. In this study, we thus attempted to investigate host- and pathogen-related factors that may influence IGRA results obtained from more than 500 patients with active TB in Viet Nam.

## Methods

### Ethics statement

A written informed consent was obtained from each participant. The study was approved by ethical committees of the Ministry of Health, Viet Nam and National Center for Global Health and Medicine, Japan respectively.

### Study population

This study is a part of our prospective study on active TB in Hanoi. After signing informed consents, 543 unrelated patients with smear-positive pulmonary TB, equal to or more than 16 years of age, and without history of TB treatment, entered this study from July 2007 to March 2009. Information of no previous TB treatment was based on self-declaration of patients and documents in district TB centers.

All had sputum smear-positive TB. Solid MTB culture on Löwenstein-Jensen media was available in 98.2% and confirmed the diagnosis in 504 patients (92.8%). The sensitivity and risk-factor analysis was made in these culture-confirmed pulmonary TB cases, although clinicians diagnosed all 543 patients as active pulmonary TB and treated them with anti-TB drugs based on the guidelines of the national TB program. Spoligotyping was used to distinguish MTB genotypes including Beijing strains [10]. At diagnosis before anti-TB treatment, the peripheral blood was drawn for testing complete blood count, HIV, IGRA and HLA genotyping. After 2 months of treatment, IGRA was tested again. Chest X-ray films were interpreted by two readers independently of IGRA results.

### IGRA

In this study, ELISA-based IGRA, QuantiFERON-TB Gold In-Tube^TM^ (QFT-IT) (Cellestis, Victoria, Australia), was used [11]. The algorithm and software (QuantiFERON-TB Gold Analysis Software, version 2.50, Cellestis) provided by the manufacturer were strictly followed for interpretation of the results [11]. The testing procedure was carefully monitored [12] and quality control of the test was done in each run, following the manufacturer's instructions. For analysis of IFN-γ values higher than 10.00 IU/ml, the truncated value (10.00 IU/ml) was used as indicated in the current software.

### HLA typing

Genomic DNA was extracted from the whole blood by using the QIAamp^TM^ DNA Blood Midi Kit (QIAGEN Sciences, Germantown, MD, USA). DNA-based HLA typing was performed by Luminex Multi-Analyte Profiling system (xMAP) with WAKFlow HLA typing kit (Wakunaga, Hiroshima, Japan) as described [13]. Briefly, highly polymorphic exon 2 of HLA-DRB1 and -DQB1 genes were amplified. Each PCR product was hybridized with sequence-specific oligonucleotide probes, complementary to the allele-specific sequences.

### Linkage disequilibrium analysis and binding peptide prediction for HLA alleles

Haploview version 4.2 (Broad Institute, Cambridge, MA) was used to calculate indicators of linkage disequilibrium, D' and r^2^, between HLA-DRB1 and -DQB1 alleles [14], [15].

To predict peptides in the protein sequence of ESAT-6, CFP10 and TB7.7 capable of binding to a given HLA-DRB1 allele *in silico*, we used the ProPred database [16], [17] with a threshold of 3%, a recommended setting.

### Statistical analysis

Factors negatively influencing IGRA results were initially screened by univariate analysis and then further investigated by multivariate analysis using a polytomous logistic regression model, with IGRA-negative and -indeterminate results as outcome variables and factors that may be involved in host immunity and disease as independent variables. Another logistic regression model using a dichotomous outcome variable, non-positive (negative and indeterminate) versus positive results, was also tested. Odd ratio (OR) and 95% confidential interval (CI) were thus calculated. HLA candidate alleles were initially screened by comparison of allele frequencies between IGRA-negative and -positive groups, and then further investigated by the logistic regression model mentioned above.

Fisher's exact test was used to detect associations. Bonferroni's correction was applied to correct multiple comparisons of association with HLA alleles. Distribution of IFN-γ values was represented by using median with interquartile range (IQR). When a value was higher than 10.00 IU/ml, truncated values (10.00 IU/ml) were presented and a quantile value based on extrapolation was supplied only as parenthetical. Wilcoxon rank-sum test and Kruskal-Wallis test were used to compare non-parametric distribution of two groups and more than two groups, respectively. *P* value <0.05 was considered to be statistically significant, unless otherwise specified. Statistical analysis was performed using Stata version 10 (StataCorp, College Station, TX).

## Results

### Characteristic of the study population

Data including QFT-IT results were analyzed in 504 new patients with culture-confirmed pulmonary TB. The median age was 38.8, the proportion of male patients was 79.2%, and HIV was positive in 8.7% of the cases. Body mass index (BMI) showed that more than 50% of the patients were underweight, following the categorization on the basis of international guidelines [18] (table 1).

**Table 1:** Characteristics of patients with smear-positive/culture-positive pulmonary tuberculosis (n = 504).

		Number	%
Age (years old) (median, IQR)		(38.8,	29.2–50.8)
Sex	Male	399	79.2
	Female	105	20.8
Body mass index	<16.0	77	15.3
	16.0–18.4	206	40.9
	18.5–24.9	218	43.2
	≥25.0	3	0.6
Smoking habit	Smoker	199	39.5
	Ex-smoker	136	27.0
	Non-smoker	168	33.3
	No answer	1	0.2
Underlying disease other than HIV*	None	435	86.3
	One	61	12.1
	More than one	8	1.6
HIV status	Positive	44	8.7
	Negative	459	91.1
	Not available	1	0.2
Lymphocyte count	≥1,000/mm3	442	87.7
	<1,000/mm3	60	11.9
	Not available	2	0.4

IQR: inter-quartile range, HIV: human immunodeficiency virus; TB: Tuberculosis;

*Includes diabetes mellitus, gastrectomy, gastric ulcer, renal failure and gout (hyperuricemia).

### QFT-IT results

In 504 patients tested, the overall sensitivity of QFT-IT was 92.3% (95% CI, 89.6%–94.4%), but decreased to 61.4% (95% CI, 45.5%–75.6%) in HIV-infected patients (table 2). The proportions of QFT-IT-negative and -indeterminate results were 4.8% (95% CI, 3.1%–7.0%) and 3.0% (95% CI, 1.7%–4.9%) respectively. All of the 15 indeterminate cases had low response to phytohaemagglutinin (PHA, or mitogen) and TB-Ag after subtracting Nil value (TBAg-Nil) (0.20 [IQR, 0.04–0.34] IU/ml and 0.03 [IQR, 0.01–0.06] IU/ml, respectively). Compared with the patients who had test-positive results, those with negative results were significantly older (median age: 48.9 [IQR, 33.2–62.6] vs 39.0 [IQR, 29.1–50.6], *P* = 0.036), and had significantly lower BMI (median BMI: 16.6 [IQR, 13.9–17.9] vs 18.3 [16.9–19.7] kg/m^2^, *P* = 0.0001) (table not shown).

**Table 2:** QFT-IT results and HIV status in smear-positive/culture-positive pulmonary TB patients.

	Positive		Negative		Indeterminate		*P* *
	n	% (95% CI)	n	% (95% CI)	n	% (95% CI)	
All (n = 504)	465	92.3 (89.6–94.4)	24	4.8 (3.1–7.0)	15	3.0 (1.7–4.9)	
HIV positive (n = 44)	27	61.4 (45.5–75.6)	5	11.4 (3.8–24.6)	12	27.3 (15.0–42.8)	<0.001
HIV negative (n = 459)	437	95.2 (92.8–97.0)	19	4.1 (2.5–6.4)	3	0.7 (0.1–1.9)	

QFT-IT: QuantiFERON-TB Gold In-Tube; HIV: human immunodeficiency virus; TB: Tuberculosis; CI: Confidence interval.

*Comparison was made between HIV-positive and HIV-negative groups.

### HLA-DRB1 and HLA-DQB1 alleles and IFN-γ responses

Since QFT-IT is based on T-cell response to MTB-specific antigenic peptides that are presented with MHC class II molecules, we investigated the role of HLA-DRB1 and -DQB1 alleles. Among seven most common HLA-DRB1 and -DQB1 alleles tested in the population [13], the allele frequencies of HLA-DRB1*0701 and DQB1*0201 in the test-negative group were significantly higher than that of the positive group (*P*<0.0001 and *P* = 0.001, respectively, which remained significant after Bonferroni's correction) (table 3).

**Table 3:** Frequencies of HLA class II alleles and QFT-IT positive/negative results.

Allele name	Number of alleles (% [95%CI])			*P* *
	Total	IGRA positive	IGRA-negative	
	2n‡ = 1008	2n = 930	2n = 48	
HLA-DRB1				
1202	337 (33.4 [30.5–36.4])	318 (34.2 [31.1–37.3])	11 (22.9 [12.0–37.3])	0.119
0901	129 (12.8 [10.8–15.0])	119 (12.8 [10.7–15.1])	6 (12.5 [4.7–25.2])	>0.999
0701	61 (6.1 [4.7–7.7])	44 (4.7 [3.5–6.3])	11 (22.9 [12.0–37.3])	<0.0001
1502	59 (5.9 [4.5–7.5])	53 (5.7 [4.3–7.4])	5 (10.4 [3.5–22.7])	0.198
0301	54 (5.4 [4.0–6.9])	49 (5.3 [3.9–6.9])	4 (8.3 [2.3–20.0])	0.324
0803	53 (5.3 [4.0–6.8])	51 (5.5 [4.1–7.1])	0 (0.0 [0.0–7.4])	0.170
1001	51 (5.1 [3.8–6.6])	49 (5.3 [3.9–6.9])	2 (4.2 [0.5–14.3])	>0.999
others	264 (26.2 [23.5–29.0])	247 (26.6 [23.7–29.5])	9 (18.8 [8.9–32.6])	0.312
HLA-DQB1				
0301	383 (38.0 [35.0–41.1])	361 (38.8 [35.7–42.0])	12 (25.0 [13.6–39.6])	0.067
0303	152 (15.1 [12.9–17.4])	138 (14.8 [12.6–17.3])	9 (18.8 [8.9–32.6])	0.414
0501	94 (9.3 [7.6–11.3])	87 (9.4 [7.6–11.4])	6 (12.5 [4.7–25.2])	0.448
0201	92 (9.1 [7.4–11.1])	74 (8.0 [6.3–9.9])	12 (25.0 [13.6–39.6])	0.001
0502	81 (8.0 [6.4–9.9])	76 (8.2 [6.5–10.1])	3 (6.3 [1.3–17.2])	>0.999
0601	70 (6.9 [5.5–8.7])	68 (7.3 [5.7–9.2])	0 (0.0 [0.0–7.4])	0.072
0401	42 (4.2 [3.0–5.6])	40 (4.3 [3.1–5.8])	1 (2.1 [0.1–11.1])	0.717
others	94 (9.3 [7.6–11.3])	86 (9.2 [7.5–11.3])	5 (10.4 [3.5–22.7])	0.797

*Comparison was made between QFT-IT-positive and -negative groups. After Bonferroni's correction, *P* <0.006 was statistically significant, considering the number of comparisons.

‡ Allele number is shown.

HLA: Human leukocyte antigen; QFT-IT: QuantiFERON-TB Gold In-Tube.

Nonrandom association between HLA-DRB1*0701 and DQB1*0202 was tested and HLA-DRB1*0701 was found to be in moderate linkage disequilibrium (LD) with HLA-DQB1*0201 allele (D' = 0.608 and r^2^ = 0.235) (table not shown). The TBAg-Nil values of IFN-γ in the HLA-DRB1*0701-negative/DQB1*0201-positive group were not significantly lower than those in the both negative group (6.65 IU/ml [IQR, 2.85–10.00 (16.06)] vs 7.76 IU/ml [IQR, 2.58–10.00 (14.87)], *P* = 0.989), whereas the IFN-γ values in the HLA-DRB1*0701-positive/DQB1*0201-negative group was significantly lower than those in the both negative group (2.30 IU/ml [IQR, 1.22–4.44] vs 7.76 IU/ml [IQR, 2.58–10.00 (14.87)], *P*<0.001) (table not shown).

TBAg-Nil values of all four patients with two HLA-DRB1*0701 alleles (homozygous for HLA-DRB1*0701) were below 0.35 IU/ml or the cutoff value; three negative, one indeterminate and none had positive results. In patients with one HLA-DRB1*0701 allele (heterozygous for HLA-DRB1*0701), proportions of negative, indeterminate and positive results were 9.4% (5/53), 7.6% (4/53) and 83.0% (44/53). In patients with no HLA-DRB1*0701 alleles (homozygous for non-HLA-DRB1*0701), the proportions were 3.6% (16/447), 2.2% (10/447) and 94.2% (421/447) respectively. Overall distribution of QFT-IT results was significantly different among HLA-DRB1*0701 genotypes (*P*<0.0001). The effect of two HLA-DRB1*0701 alleles on QFT-IT negativity was significant (3/4 vs 16/447, *P* = 0.0002), whereas the effect of one HLA-DRB1*0701 allele on QFT-IT negativity was weaker than that of two HLA-DRB1*0701 alleles (5/53 vs 3/4, *P* = 0.007) and did not reach significant levels (5/53 vs 16/447, *P* = 0.06) when “no alleles” was regarded as a category for reference purposes.

**Figure 1:**
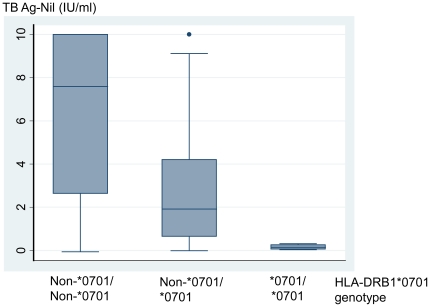
IFN-γ response to TB antigens stratified by HLA-DRB1*0701 genotypes in smear-positive/culture-positive pulmonary TB patients (n = 504). HLA-DRB1*0701 genotype significantly affected TB-Ag specific IFN-γ response (TBAg-Nil) (*P*<0.001). IFN: Interferon; TB: Tuberculosis; HLA: Human leukocyte antigen; Ag: Antigen.

Distribution of IFN-γ values may provide information about the mechanism by which false negative results are observed. We reviewed the relationship between IFN-γ values and HLA-DRB1*0701 genotypes (Figure 1). HLA-DRB1*0701 genotype significantly affected TB-Ag specific IFN-γ response (TBAg-Nil) (*P*<0.001): The IFN-γ values in patients with two HLA-DRB1*0701 alleles (homozygous for HLA-DRB1*0701) were significantly lower than those in patients with one HLA-DRB1*0701 allele (heterozygous for HLA-DRB1*0701) (0.15 IU/ml [IQR, 0.06–0.26] vs 1.91 IU/ml [IQR, 0.65–4.21], *P* = 0.008). As a reference, the median of IFN-γ values in patients who did not bear any HLA-DRB1*0701 alleles was 7.59 IU/ml [IQR, 2.63–10.00 (14.92)].

No association was clinically observed between HLA-DRB1*0701-containing genotype and disease severity assessed by either cavity or infiltrate on CXR respectively (data not shown). QFT-IT test was performed again after two months of anti-TB treatment in 17 out of 19 HIV-negative patients with QFT-IT-negative results. All 7 patients who carried one or two HLA-DRB1*0701 alleles showed negative results again, whereas it remained negative only in 6 out of 10 patients without carrying the HLA allele, though this difference did not reach significant levels (*P* = 0.103).

Analysis of 51 HLA-DR alleles registered on the ProPred database revealed that the average number of epitopes predicted in the overall amino acid sequences of ESAT-6 (95 amino acids), CFP10 (100 amino acids) and TB7.7 p4 (18 amino acids) to bind a given allele was median of 4 with IQR in 3 to 5, but the number of epitopes predicted for HLA-DRB1*0701 was only one (data not shown).

### Univariate analysis

Host factors including age, sex, BMI, underlying diseases, disease status, and inherent characteristics of pathogen were analyzed. The number of HLA-DRB1*0701 alleles carried by the patients appeared to be associated with the test-negative results. For this reason, this variable was also included in the statistical model.

In univariate analysis, increased age by year, BMI<16.0, HIV co-infection and the number of HLA-DRB1*0701 alleles carried by patients showed significant associations with the negative results (OR = 1.04 [95%CI, 1.01–1.06], 7.27 [95%CI, 2.17–24.38], 4.26 [95%CI, 1.48–12.28], and 5.47 [95%CI, 2.58–11.61] respectively) (table 4). Sex, underlying diseases other than HIV infection, hospitalization, presence of infiltrates in more than half of the lung field and cavitary lesions on chest X-ray (CXR) did not show significant associations (data not shown). Beijing MTB strains were less frequently seen in the test-negative group (OR = 0.29 [95% CI, 0.11–0.76]). Multi-drug resistant (MDR)-TB strains showed no association with IGRA-negative results.

**Table 4:** Univariate analysis using polytomous logistic regression model for factors associated with QFT-IT-negative and -indeterminate results (n = 503).

		QFT-IT-negative results			QFT-IT-indeterminate results		
		Proportion (%)	OR*	95% CI	Proportion (%)	OR*	95% CI
Sex	Male	21/398 (5.3)	1.00		13/398 (3.3)	1.00	
	Female	3/105 (2.9)	0.52	0.15–1.78	2/105 (1.9)	0.56	0.12–2.52
Age (years)			1.04	1.01–1.06		0.98	0.94–1.02
BMI	18.5–24.9	4/217 (1.8)	1.00		3/217 (1.4)	1.00	
	<16.0	9/77 (11.7)	7.27	2.17–24.38	3/77 (3.9)	3.23	0.64–16.40
	16.0–18.5	11/206 (5.3)	3.10	0.97–9.92	9/206 (4.4)	3.39	0.90–12.70
	≥25.0	0/3 (0.0)	NA	NA	0/3 (0.0)	NA	NA
Underlying condition	None	20/434 (4.6)	1.00		14/434 (3.2)	1.00	
	One	4/61 (6.6)	1.43	0.47–4.34	1/61 (1.6)	0.51	0.07–3.96
	More than one	0/8 (0.0)	NA	NA	0/8 (0.0)	NA	NA
HIV status	Negative	19/459 (4.1)	1.00		3/459 (0.7)	1.00	
	Positive	5/44 (11.4)	4.26	1.48–12.28	12/44 (27.3)	64.74	17.23–243.20
Lymphocyte count (cells/mm3)	≥1,000	19/441 (4.3)	1.00		4/441 (0.9)	1.00	
	<1,000	5/60 (8.3)	2.51	0.89–7.04	11/60 (18.3)	26.19	8.00–85.72
Direct smear result	Scanty	3/65 (4.6)	1.00		3/65 (4.6)	1.00	
	1+ and more	21/438 (4.8)	1.02	0.30–3.52	12/438 (2.7)	0.58	0.16–2.13
Cavity on CXR	No	6/145 (4.1)	1.00		9/145 (6.2)	1.00	
	Yes	16/327 (4.9)	1.13	0.43–2.95	4/327 (1.2)	0.19	0.06–0.62
Infiltrate in >3 lung zones	No	15/391 (3.8)	1.00		12/391 (3.1)	1.00	
	Yes	7/83 (8.4)	2.26	0.89–5.75	1/83 (1.2)	0.40	0.05–3.16
Hospitalization	No	17/375 (4.5)	1.00		9/375 (2.4)	1.00	
	To TB ward	5/104 (4.8)	1.10	0.40–3.07	6/104 (5.8)	2.50	0.87–7.21
	To ER	2/24 (8.3)	1.87	0.41–8.59	0/24 (0.0)	NA	NA
HLA-DRB1*0701 (the number of alleles)			5.47†	2.58–11.61		4.66†	1.83–11.88
MDR	No	21/466 (4.5)	1.00		12/466 (2.6)	1.00	
	Yes	2/22 (9.1)	2.43	0.53–11.19	3/22 (13.6)	6.37	1.64–24.68
MTB strain	Non-Beijing	17/229 (7.4)	1.00		8/229 (3.5)	1.00	
	Beijing	6/259 (2.3)	0.29	0.11–0.76	7/259 (2.7)	0.73	0.26–2.03

QFT-IT: QuantiFERON-TB Gold In-Tube; BMI: Body mass index; CXR: Chest X-ray; MDR: Multi drug resistance; TB: Tuberculosis; ER: Emergency room; MTB: *Mycobacterium tuberculosis;* CI: Confidence interval; NA: Not available.

*OR: Multinomial odds ratio, also known as relative risk ratio, that is obtained by exponentiating the logit coefficient.

† OR per unit change in the number of alleles: Distribution of QFT-IT results and the number of HLA-DRB1*0701 alleles was shown in the text.

With QFT-IT indeterminate results, HIV co-infection, low lymphocyte count, MDR and the number of HLA-DRB1*0701 alleles showed significant associations (OR = 64.74 [95% CI, 17.23–243.20], 26.19 [95% CI, 8.00–85.72], 6.37 [95% CI, 1.64–24.68] and 4.66 [95% CI, 1.83–11.88], respectively).

### Multivariate analysis

Age, sex, BMI, HIV status, lymphocyte count, and the number of HLA-DRB1*0701 alleles were put into the initial model for multivariate analysis. MTB strain and MDR-TB were not put together into this model because of the considerable number of missing values but analyzed separately as described later. In the final model, increased age by year, BMI <16.0, HIV co-infection and the number of HLA-DRB1*0701 alleles showed significant association with QFT-IT negativity (OR = 1.04 [95% CI, 1.01–1.07], 5.42 [95% CI, 1.48–19.79], 6.38 [95% CI, 1.78–22.92] and 5.09 [95% CI, 2.31–11.22] respectively) (table 5).

**Table 5:** Multivariate analysis using polytomous logistic regression model for factors associated with QFT-IT-negative and -indeterminate results (n = 503).

		QFT-IT-negative results			QFT-IT-indeterminate results		
		Proportion (%)	OR*	95% CI	Proportion (%)	OR*	95% CI
Age (years)			1.04	1.01–1.07		1.04	0.97–1.11
BMI	18.5–24.9	4/217 (1.8)	1.00		3/217 (1.4)	1.00	
	<16.0	9/77 (11.7)	5.42	1.48–19.79	3/77 (3.9)	1.82	0.29–11.18
	16.0–18.5	11/206 (5.3)	2.65	0.79–8.85	9/206 (4.4)	1.92	0.43–8.48
	≥25.0	0/3 (0.0)	NA	NA	0/3 (0.0)	NA	NA
HIV status	Negative	19/459 (4.1)	1.00		3/459 (0.7)	1.00	
	Positive	5/44 (11.4)	6.38	1.78–22.92	12/44 (27.3)	99.59	15.85–625.61
HLA-DRB1*0701 (the number of alleles)			5.09†	2.31–11.22		4.25†	1.27–14.16

QFT-IT: QuantiFERON-TB Gold In-Tube; BMI: Body mass index; HIV: human immunodeficiency virus; CI: Confidence interval; NA: Not available

*OR: Multinomial odds ratio, also known as relative risk ratio, that is obtained by exponentiating the logit coefficient.

† OR per unit change in the number of alleles: Distribution of QFT-IT results and the number of HLA-DRB1*0701 alleles was shown in the text.

HIV co-infection and the number of HLA-DRB1*0701 alleles were also significantly associated with QFT-IT indeterminate results (OR = 99.59 [95% CI, 15.85–625.61] and 4.25 [95% CI, 1.27–14.16] respectively).

When non-positive (negative and indeterminate) results of QFT-IT were compared with positive results, increased age by year, BMI <16.0, HIV co-infection and the number of HLA-DRB1*0701 alleles showed similarly high odds ratios (Table S1).

### Bacterial characteristics and IFN-γ responses

Among 488 patients for whom information of QFT-IT and MTB strains were both available, concentrations of IFN-γ responding to MTB-specific antigens were neither different between patient groups with Beijing and non-Beijing MTB strains (6.92 IU/ml [2.19–10.00 (14.42)] vs 6.00 IU/ml [2.12–10.00 (14.54)]) nor between patient groups with MDR-TB and non-MDR-TB strains (4.19 IU/ml [0.62–10.00 (15.72)] vs 6.57 IU/ml [2.19–10.00 (14.47)]) (table not shown).

## Discussion

We calculated the test sensitivity of ELISA-based IGRA among active TB patients in Viet Nam and made an extensive analysis of the factors associated with the false-negative results, which include increased age by year, extremely low BMI, HIV co-infection, and the number of HLA-DRB1*0701 alleles carried by the patients.

Aging is known as a risk factor for false-negative results [19], [20]. Kobashi et al. [19] reports that the positive rate for both ESAT-6 and CFP-10 antigens of QuantiFERON TB-2G tested in the patients ≥80 years old is significantly lower than that in younger patients. In another study conducted by Liao et al. [20], using ELISPOT assay, increasing age is associated with false-negative results. HIV co-infection was associated with indeterminate results as well as false-negative results, presumably due to strong suppression of mitogenic response [21].

Severe wasting disease or malnutrition causes unhealthy emaciation with extremely low BMI, debilitating the patients and also suppressing systemic immune response [22]. In our study, BMI <16.0 kg/m^2^, was significantly associated with IGRA negativity whereas moderate and mild underweight (BMI from 16.0 to less than 18.5) were not. The proportion of BMI <18.5 in the general population in Hanoi was only 13.3% [23], indicating that very low BMI in our study population is associated with active TB disease. However, it is not known whether this emaciation is observed mainly as a result of the current wasting disease or partly a risk factor for disease development.

In this study, we newly demonstrated that a particular MHC class II allele, HLA-DRB1*0701, was strongly associated with low TBAg-Nil values observed in indeterminate and negative results. HLA-DRB1*0701-positive/DQB1*0201-negative group but not HLA-DRB1*0701-negative/DQB1*0201-positive group suppressed the IFN-γ response, which suggests that HLA-DQB1*0701, but not HLA-DQB1*0201 has a primary role. The negative effect of HLA-DRB1*0701 on the IFN-γ values appeared to intensify in proportion to the number of HLA-DRB1*0701 alleles. The association between the increased number of the HLA alleles and QFT-IT negative results was demonstrated by the analysis using a logistic regression model.

After two months of anti-TB treatment, all of our IGRA-negative patients bearing the HLA allele continued to show negative IGRA results. There was no significant association between the extent of disease on CXR and the HLA-DRB1*0701 genotype (data not shown), suggesting that the allele does not seem to affect the assay results through modulation of disease severity. *In silico* analysis suggested the low affinity of HLA-DRB1*0701 in binding with both ESAT-6 and CFP10 epitopes, and possibly failing to present them to T-cells for initiation of Th1 immune response efficiently [24].

Considering the low frequency of HLA-DRB1*0701 in the population tested, this finding may not have major clinical implications. However, we should bear in mind that negative QFT-IT results might be experienced in TB-infected individuals within a certain genetic background of the host even without apparent cause of immunodeficiency. In addition, it might be necessary to be investigated carefully in Southwestern Europe, North Africa, East Sub-Saharan Africa, West and South Asia among others, where high frequency (>15%) of the allele has been reported [25] and more than 2% of the people are supposed to possess this allele as homozygote. Further clinical investigations about HLA type and IGRA and *in vitro* experiments would contribute to a better understanding of IGRA performance in general and of QFT-IT in particular.

In analogy with negative results of tuberculin skin testing occasionally obtained in severe TB disease [26], IGRA-false-negative results may be caused by inefficient activation of antigen-specific CD4 T-cells [27], based on poorly-defined regulatory mechanism [28], [29]. T-cell trafficking to the active TB sites or compartmentalization may also be involved in the suppressive response in circulating blood [28]. However, this mechanism may not explain a major part of false-negative results in our study because the extent of infiltrates or presence of cavity on CXR did not show significant effects on the assay results.

Beijing MTB strains have spread rapidly in Asia and previous reports show that these are more adapted to the human body evading immune mechanism than others [30]. Although an inverse association was apparently observed between isolation of Beijing strains and IGRA negativity in our study, it may be attributed to unknown factors we could not access, since we made no demonstration of difference in TBAg-induced IFN-γ levels between Beijing and non-Beijing strains.

The overall sensitivity of QFT-IT in our population was considerably high among high TB burden countries from Cape Town in South Africa, the Gambia, Zambia, India, and some other countries [3]. This seems to be due to the lower proportion of false-negative results in our study (4.8%) compared to (9.1% to 29%) in those studies. Several possible reasons for the interpretation of this point derive from our findings and others [19], [20]: low proportion of underlying diseases including HIV, very few patients receiving immunosuppressive therapy, and recruitment of only new patients with sputum smear-confirmed pulmonary TB.

Our study had some limitations. Firstly, a clinical laboratory to measure CD4 count was not accessible during the study period, although CD4 count is an important parameter for this assessment [31]. Decrease in total lymphocyte count was used as a surrogate marker. Secondly, only smear-positive patients without previous treatment have been recruited, which may not allow us to generalize our results to all types of TB. Thirdly, further investigation is necessary to know whether all of the factors identified here affect results of ELISPOT-based IGRA as well. Lastly, the number of patients showing negative results was rather small despite the large number of recruited patients in our study. This is a limitation to analyze statistical significance in general. However, we were able to identify a novel host genetic factor, HLA*DRB1-0701. If well-known factors such as HIV co-infection were predominant in the studied population, individuals bearing the host genetic factor might have a chance of having those extrinsic factors together and it might be difficult to demonstrate that their genetic difference is a primary cause of false negativity.

Although some of the factors associated with IGRA-negative results have been proposed or even studied adopting a piecemeal method [2], [3], the strong point of our study is that effects of all factors have been evaluated simultaneously by using appropriate statistical models, which provided a comprehensive insight into this area of interest.

In conclusion, we identified a specific HLA class II allele and characterized a variety of factors that possibly lead to false negativity of IGRA in active pulmonary TB. Detailed investigation of these unfavorable factors is necessary and would help to understand further the performance of the assay.

## Supporting Information

Table S1 **Univariate and multivariate analysis using logistic regression model for factors associated with QFT-IT non-positive (negative and indeterminate) results (n = 503).**
(DOC)

## References

[1] WHO Global Tuberculosis Control (2010). http://whqlibdoc.who.int/publications/2010/9789241564069_eng.pdf.

[2] Pai M, Menzies D (2007). Interferon-ã release assays: what is their role in the diagnosis of active tuberculosis?. Clin Infect Dis.

[3] Dheda K, van Zyl Smit R, Badri M, Pai M (2009). T-cell interferon-γ release assays for the rapid immunodiagnosis of tuberculosis: clinical utility in high-burden vs. low-burden settings.. Curr Opin Pulm Med.

[4] Diel R, Loddenkemper R, Nienhaus A (2010). Evidence-based comparison of commercial interferon-γ release assays for detecting active TB: a metaanalysis.. Chest.

[5] European Centre for Disease Prevention and Control (2011). Use of interferon-gamma release assays in support of TB diagnosis.. http://ecdc.europa.eu/en/publications/Publications/1103_GUI_IGRA.pdf.

[6] Centers for Disease Control and Prevention (2010). Updated Guidelines for Using Interferon Gamma Release Assays to Detect *Mycobacterium tuberculosis* Infection — United States.. http://www.cdc.gov/mmwr/pdf/rr/rr5905.pdf.

[7] Sester M, Sotgiu G, Lange C, Giehl C, Girardi E (2011). Interferon-γ release assays for the diagnosis of active tuberculosis: a systematic review and meta-analysis.. Eur Respir J.

[8] Ling DI, Pai M, Davids V, Brunet L, Lenders L (2011). Are interferon-{gamma} release assays useful for active tuberculosis in a high-burden setting?. Eur Respir J.

[9] Mustafa AS, Oftung F, Amoudy HA, Madi NM, Abal AT (2000). Multiple epitopes from the *Mycobacterium tuberculosis* ESAT-6 antigen are recognized by antigen-specific human T cell lines.. Clin Infect Dis.

[10] Brudey K, Driscoll JR, Rigouts L, Prodinger WM, Gori A (2006). *Mycobacterium tuberculosis* complex genetic diversity: mining the fourth international spoligotyping database (SpolDB4) for classification, population genetics and epidemiology.. BMC Microbiol.

[11] Cellestis website QuantiFERON®-TB Gold In-Tube.. http://www.cellestis.com/IRM/Content/usa/qftproducts_tbgoldintube.html.

[12] Hang NT, Ishizuka N, Keicho N, Hong LT, Tam DB (2009). Quality assessment of an interferon-γ release assay for tuberculosis infection in a resource-limited setting.. BMC Infect Dis.

[13] Hoa BK, Hang NT, Kashiwase K, Ohashi J, Lien LT (2008). HLA-A, -B, -C, -DRB1 and -DQB1 alleles and haplotypes in the Kinh population in Vietnam.. Tissue Antigens.

[14] Broad Institute website Haploview.. http://www.broadinstitute.org/scientific-community/science/programs/medical-and-population-genetics/haploview/haploview.

[15] Barrett JC, Fry B, Maller J, Daly MJ (2005). Haploview: analysis and visualization of LD and haplotype maps.. Bioinformatics.

[16] Bioinformatics Center IMTECH website Propred. MHC Class-II Binding Peptide Prediction Server.. http://www.imtech.res.in/raghava/propred.

[17] Singh H, Raghava GP (2001). ProPred: prediction of HLA-DR binding sites.. Bioinformatics.

[18] WHO Expert Consultation (2004). Appropriate body-mass index for Asian populations and its implications for policy and intervention strategies.. Lancet.

[19] Kobashi Y, Mouri K, Yagi S, Obase Y, Miyashita N, Okimoto N (2008). Clinical utility of the QuantiFERON TB-2G test for elderly patients with active tuberculosis.. Chest.

[20] Liao CH, Lai CC, Tan CK, Chou CH, Hsu HL, Tasi TH (2009). False-negative results by enzyme-linked immunospot assay for interferon-gamma among patients with culture-confirmed tuberculosis.. J Infect.

[21] Syed Ahamed Kabeer B, Sikhamani R, Swaminathan S, Perumal V, Paramasivam P (2009). Role of interferon γ release assay in active TB diagnosis among HIV infected individuals.. PLoS One.

[22] Schluger NW, Rom WN (1998). The host immune response to tuberculosis.. Am J Respir Crit Care Med.

[23] Walls HL, Peeters A, Son PT, Quang NN, Hoai NT, Loi do D (2009). Prevalence of underweight, overweight and obesity in urban Hanoi, Vietnam.. Asia Pac J Clin Nutr.

[24] Arend SM, Geluk A, van Meijgaarden KE, van Dissel JT, Theisen M (2000). Antigenic equivalence of human T-cell responses to *Mycobacterium tuberculosis*-specific RD1-encoded protein antigens ESAT-6 and culture filtrate protein 10 and to mixtures of synthetic peptides.. Infect Immun.

[25] dbMHC IHWG Projects website Anthropology. Class II Allele Frequencies.. http://www.ncbi.nlm.nih.gov/projects/gv/mhc/ihwg.cgi?cmd=page&page=AnthroMain.

[26] Diagnostic Standards and Classification of Tuberculosis in Adults and Children (2000). Am J Respir Crit Care Med.

[27] Pathan AA, Wilkinson KA, Klenerman P, McShane H, Davidson RN (2001). Direct ex vivo analysis of antigen-specific IFN-ã-secreting CD4 T cells in *Mycobacterium tuberculosis*-infected individuals: associations with clinical disease state and effect of treatment.. J Immunol.

[28] Jo EK, Park JK, Dockrell HM (2003). Dynamics of cytokine generation in patients with active pulmonary tuberculosis.. Curr Opin Infect Dis.

[29] Dheda K, Schwander SK, Zhu B, van Zyl-Smit RN, Zhang Y (2010). The immunology of tuberculosis: from bench to bedside.. Respirology.

[30] Parwati I, van Crevel R, van Soolingen D (2010). Possible underlying mechanisms for successful emergence of the *Mycobacterium tuberculosis* Beijing genotype strains.. Lancet Infect Dis.

[31] Lugos MD, Adetifa IM, Donkor S, Hill PC, Adegbola RA (2009). Evaluation of the contribution of major T cell subsets to IFN-gamma production in TB infection by ELISPOT.. Immunol Invest.

