# Supplementary material for: Analysis of Factors Lowering Sensitivity of Interferon-γ Release Assay for Tuberculosis
Source: PLoS One. 2011 Aug 19;6(8):e23806. doi: 10.1371/journal.pone.0023806 (PMC3158775; doi:10.1371/journal.pone.0023806)
Supplement: Table S1 — Univariate and multivariate analysis using logistic regression model for factors associated with QFT-IT non-positive (negative and indeterminate) results (n = 503). (DOC) [file pone.0023806.s001.doc]

**Table S1:** Univariate and multivariate analysis using logistic regression model for factors associated with QFT-IT non-positive (negative and indeterminate) results (n = 503).

|  |  | **Proportion (%)** | **Univariate analysis** | | **Multivariate analysis**† | |
| --- | --- | --- | --- | --- | --- | --- |
|  |  |  | **OR*** | **95% CI** | **OR*** | **95% CI** |
| Sex | Male | 34/398 (8.5) | 1.00 |  |  |  |
|  | Female | 5/105 (4.8) | 0.54 | 0.20 - 1.40 |  |  |
| Age (years) |  |  | 1.02 | 1.00 - 1.04 | 1.04 | 1.01 - 1.07 |
| BMI | 18.5 - 24.9 | 7/217 (3.2) | 1.00 |  | 1.00 |  |
|  | < 16.0 | 12/77 (15.6) | 5.54 | 2.09 - 14.65 | 3.88 | 1.31- 11.49 |
|  | 16.0 - 18.5 | 20/206 (9.7) | 3.23 | 1.33 - 7.80 | 2.35 | 0.90 - 6.15 |
|  | ≥ 25.0 | 0/3 (0.0) | NA | NA | NA | NA |
| Underlying condition | None | 34/434 (7.8) | 1.00 |  |  |  |
|  | One | 5/61 (8.2) | 1.05 | 0.39 - 2.80 |  |  |
|  | More than one | 0/8 (0.0) | NA | NA |  |  |
| HIV status | Negative | 22/459 (4.8) | 1.00 |  | 1.00 |  |
|  | Positive | 17/44 (38.6) | 12.51 | 5.95 - 26.28 | 20.10 | 7.56 - 53.46 |
| Lymphocyte count (cells/mm3) | ≥ 1,000 | 23/441 (5.2) | 1.00 |  |  |  |
|  | < 1,000 | 16/60 (26.7) | 6.61 | 3.25 - 13.44 |  |  |
| Direct smear result | Scanty | 6/65 (9.2) | 1.00 |  |  |  |
|  | 1+ and more | 33/438 (7.5) | 0.80 | 0.32 - 1.99 |  |  |
| Cavity on CXR | No | 15/145 (10.3) | 1.00 |  |  |  |
|  | Yes | 20/327 (6.1) | 0.56 | 0.28 - 1.14 |  |  |
| Infiltrate in >3 lung zones | No | 27/391 (6.9) | 1.00 |  |  |  |
|  | Yes | 8/83 (9.6) | 1.44 | 0.63 - 3.29 |  |  |
| Hospitalization | No | 26/375 (6.9) | 1.00 |  |  |  |
|  | To TB ward | 11/104 (10.6) | 1.59 | 0.76 - 3.33 |  |  |
|  | To ER | 2/24 (8.3) | 1.22 | 0.27 - 5.48 |  |  |
| HLA-DRB1*0701 (the number of alleles) |  |  | 5.14‡ | 2.71 - 9.75 | 4.79‡ | 2.34 - 9.79 |
| MDR | No | 33/465 (7.1) | 1.00 |  |  |  |
|  | Yes | 5/22 (22.7) | 3.85 | 1.33 - 11.09 |  |  |
| MTB strain | Non-Beijing | 25/228 (11.0) | 1.00 |  |  |  |
|  | Beijing | 13/259 (5.0) | 0.43 | 0.21 - 0.86 |  |  |

OR: odds ratio; QFT-IT: QuantiFERON-TB Gold In-Tube; BMI: Body mass index; CXR: Chest X-ray; MDR: Multi drug resistance; TB: Tuberculosis; ER: Emergency room; MTB: *Mycobacterium tuberculosis;* CI: Confidence interval; NA: Not available.

* OR for non-positive versus positive results.

† CXR-related variables and other variables, such as *Mycobacterium tuberculosis*’s strain and status of multidrug-resistance, were not included in the model due to a number of missing values.

‡ OR per unit change in the number of alleles.
